# Functional heterogeneity in the left lateral posterior parietal cortex during visual and haptic crossmodal dot‐surface matching

**DOI:** 10.1002/brb3.2033

**Published:** 2021-01-19

**Authors:** Jiajia Yang, Yinghua Yu, Hiroaki Shigemasu, Hiroshi Kadota, Kiyoshi Nakahara, Takanori Kochiyama, Yoshimichi Ejima, Jinglong Wu

**Affiliations:** ^1^ Graduate School of Interdisciplinary Science and Engineering in Health Systems Okayama University Okayama Japan; ^2^ Section on Functional Imaging Methods National Institute of Mental Health Bethesda MD USA; ^3^ Center for Information and Neural Networks National Institute of Information and Communications Technology Suita Japan; ^4^ Kochi University of Technology Kochi Japan; ^5^ ATR Brain Activity Imaging Center Kyoto Japan; ^6^ Beijing Institute of Technology Beijing China

**Keywords:** crossmodal processing, fMRI, haptic dot‐surface matching, lateral posterior parietal cortex, memory retrieval

## Abstract

**Background:**

Vision and touch are thought to contribute information to object perception in an independent but complementary manner. The left lateral posterior parietal cortex (LPPC) has long been associated with multisensory information processing, and it plays an important role in visual and haptic crossmodal information retrieval. However, it remains unclear how LPPC subregions are involved in visuo‐haptic crossmodal retrieval processing.

**Methods:**

In the present study, we used an fMRI experiment with a crossmodal delayed match‐to‐sample paradigm to reveal the functional role of LPPC subregions related to unimodal and crossmodal dot‐surface retrieval.

**Results:**

The visual‐to‐haptic condition enhanced the activity of the left inferior parietal lobule relative to the haptic unimodal condition, whereas the inverse condition enhanced the activity of the left superior parietal lobule. By contrast, activation of the left intraparietal sulcus did not differ significantly between the crossmodal and unimodal conditions. Seed‐based resting connectivity analysis revealed that these three left LPPC subregions engaged distinct networks, confirming their different functions in crossmodal retrieval processing.

**Conclusion:**

Taken together, the findings suggest that functional heterogeneity of the left LPPC during visuo‐haptic crossmodal dot‐surface retrieval processing reflects that the left LPPC does not simply contribute to retrieval of past information; rather, each subregion has a specific functional role in resolving different task requirements.

## Graphical abstract

Our findings have provided the knowledge that left lateral posterior parietal cortex subregions do not merely contribute to retrieving past information; rather, each subregion has a more specific functional role in resolving crossmodal retrieval requirements.

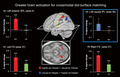

## 1 INTRODUCTION

Humans can effortlessly recognize objects using different sensory modalities (e.g., see or touch a tennis ball). This suggests that information about an object produced by different sensory modalities converges somewhere in the human brain to form representations that are invariant to the input sensory modality. The lateral posterior parietal cortex (LPPC) plays a pivotal role in memory retrieval (Sestieri et al., 2017) and has long been associated with multisensory information convergence and divergence (Meyer & Damasio, 2009; Stein & Stanford, 2008; Whitaker et al., 2008). However, the fundamental question of how the LPPC contributes to crossmodal memory retrieval is still unresolved.

The contribution of the LPPC to memory retrieval is typically strong in the left hemisphere (Guerin & Miller, 2009; Hutchinson et al., 2009), and subregions of the LPPC are characterized by distinct functional properties during memory retrieval (Nelson et al., 2010; Sestieri et al., 2011). Specifically, the left intraparietal sulcus (IPS) has been implicated in processes related to familiarity judgments and attentional control independent of sensory modalities or task parameters (Hutchinson et al., 2014; Nelson et al., 2010). Activation in the posterior part of the left inferior parietal lobule (IPL) mostly reflects recollection of specific details from the encoding phase (Sestieri et al., 2017), whereas the anterior superior parietal lobule (SPL) plays an important role in the manipulation and rearrangement of information in working memory (Koenigs et al., 2009). Thus, this evidence suggests that both functional and anatomical segregations of the LPPC are involved in unimodal memory retrieval. However, the potential intrinsic differences of the LPPC in the contributions to crossmodal memory retrieval have not been addressed to date.

To identify the brain function underlying crossmodal processing, a crossmodal delayed match‐to‐sample (DMS) paradigm has been used in neuroimaging studies (Kassuba et al., 2013; Lacey & Sathian, 2014; Tal & Amedi, 2009). This paradigm allowed us to explore the brain network during sample stimulus encoding of each modality and then discuss how the brain network changed during the retrieval of sample features from another sensory modality relative to unimodal memory retrieval. In the visual and haptic systems, for stimulus encoding processing, previous studies demonstrated that both systems share a large proportion the process of extracting an object that potentially raised common neural substrates in the human brain (Kitada et al., 2006; Masson et al., 2016; Sciutti et al., 2019). In contrast, the brain network responsible for stimulus matching, even for unimodal conditions, showed more variation, and sometimes it depended on the task requirement (Eck et al., 2016). One possibility is that people do not aim to obtain the stimuli's general features during the matching phase while obtaining information to directly evaluate whether the stimulus matches the encoded stimulus to support their decision. Furthermore, the modality‐specific encoding strategy is also a factor that contributes to match processing. For instance, the haptic system is involved in encoding the surface substance rather than shape compared with the visual system (Picard, 2006), and such a difference may directly influence memory retrieval processing during the crossmodal matching phase. Therefore, a well‐designed DMS paradigm should provide more definite evidence to reveal the functional role of crossmodal memory retrieval areas such as the left LPPC.

In the present study, to reveal how crossmodal memory retrieval modulates brain activity in the left LPPC, we performed a crossmodal visuo‐haptic dot‐surface‐matching functional magnetic resonance imaging (fMRI) experiment. We designed four dot‐surface‐matching conditions: two unimodal (visual‐to‐Visual, vV; haptic‐to‐Haptic, hH) and two crossmodal (haptic‐to‐Visual, hV; visual‐to‐Haptic, vH) conditions. The subjects were allowed to touch or see a dot‐surface during the encoding phase, while they did not know whether they had to match this dot‐surface by using the same or a different sensory modality before the matching phase instruction was presented. Since dot‐surface perception weakly enables humans to determine whether the touched surface and the seen surface are the same, we asked the subjects to find the stimuli with the same or most similar dot‐surface from the five stimuli during the matching phase. This approach has the advantage of maintaining the constant encoding processing of unimodal and crossmodal conditions. Concurrently, it also allowed us to assess the crossmodal memory retrieval modulation in the left LPPC by contrasting the matching phase of crossmodal conditions (hV and vH) to unimodal conditions (vV and hH).

## 2 MATERIALS AND METHODS

### 2.1 Subjects

Eighteen healthy right‐handed subjects (14 males and four females; age 21–26 years, mean age 21.9 ± 0.3 years) participated in the fMRI experiment. None of the subjects reported any loss of tactile sensation or a history of major medical or neurological illness, such as epilepsy, significant head trauma, or alcohol dependence. The experimental protocol was approved by the local Medical Ethics Committee at Okayama University Hospital and the Kochi University of Technology. All subjects provided written informed consent prior to participation in this study.

### 2.2 Stimuli

**FIGURE 1:**
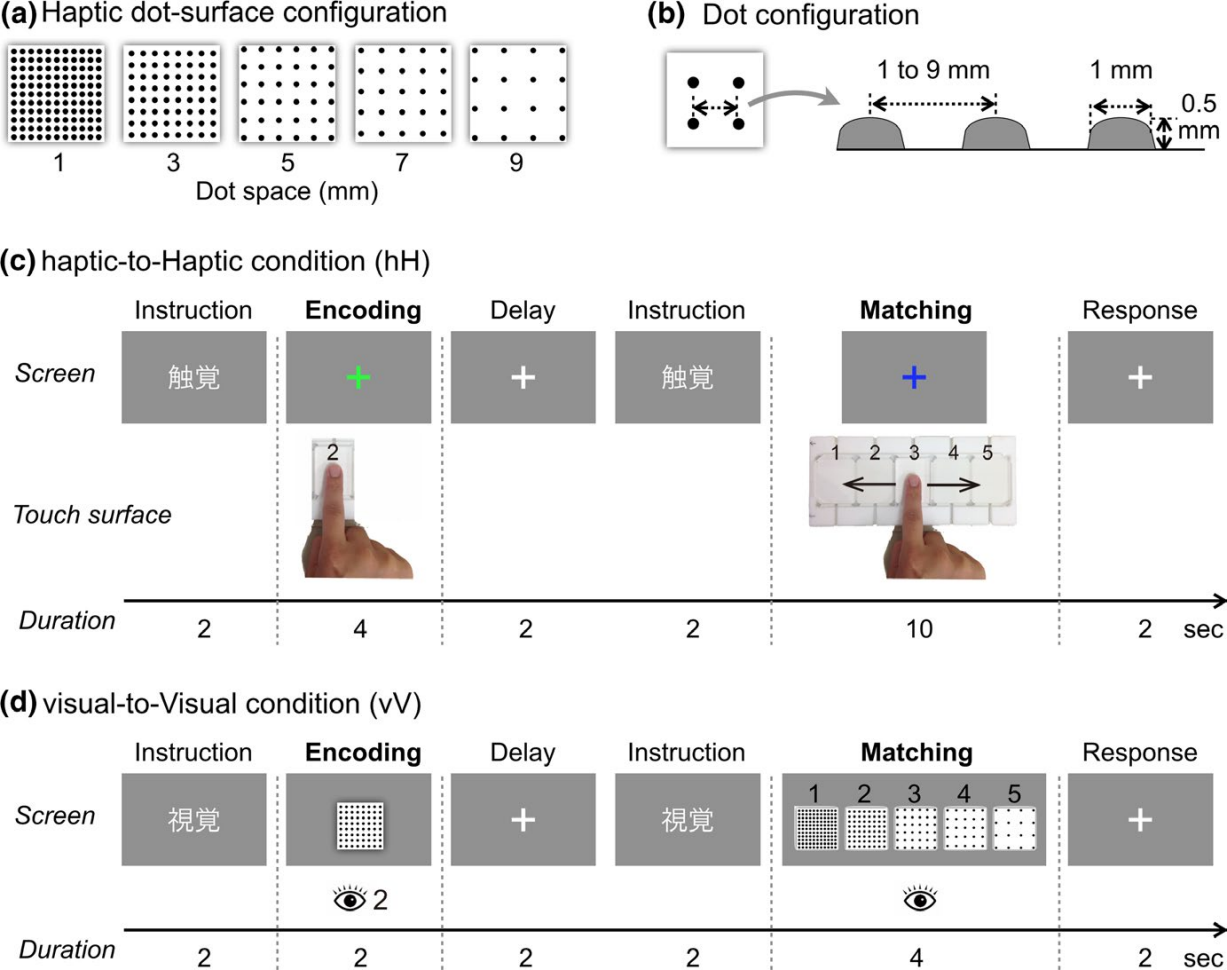
The configuration of (a) the haptic dot‐surfaces. The haptic dot‐surfaces consisted of rectangular arrays of dots with an identical spatial period (1–9 mm, distance between the centers of adjacent dots). (b) The hemispheroidal raised dots consisted of custom‐built plastic shapes raised by 0.5 mm and a 1.0 mm diameter on the bottom. (c) Illustration of one trial of the haptic unimodal condition. First, subjects fixated on the visual screen. Each trial started with 2 s of visual instructions (i.e., the Chinese characters for “haptic”). After the instructions, a green fixation cross was presented, and subjects were asked to explore the first haptic dot‐surface for 4 s. Next, following a 2‐s delay, matching phase modality instructions were presented for 2 s. After that, a blue fixation cross was presented, and subjects were asked to explore five haptic dot‐surfaces for 10 s. Subsequently, subjects were asked to indicate which of the five surfaces was most similar to the first surface using the response keys. (d) The visual–visual condition used the same procedure, but the stimuli duration and instructions were adjusted

In the present study, we designed a series of raised dot‐surfaces with different dot‐spacing based on the previous tactile/haptic texture perception studies (Bourgeon et al., 2016; Dépeault et al., 2009; Yang et al., 2017, 2020). Figure 1a shows the illustration of five kinds of dot‐surfaces used in the experiment, which consisted of rectangular arrays of hemispheroidal raised dots with an identical distance between the centers of adjacent dots in each row. The interdot spacing ranged from 1.0 to 9.0 mm and increased in steps of 2.0 mm (i.e., 1, 3, 5, 7, and 9 mm). As shown in Figure 1b, the hemispheroidal raised dots consisted of custom‐built plastic shapes raised by 0.5 mm from a 40.0 × 50.0 mm rectangle base and a 1.0 mm diameter on the bottom. The dot‐surfaces were affixed to a customized plastic case that consisted of five rectangular spaces. We designed two cover plates: one allowed subject to touch only one dot‐surface during the encoding phase, and the second allowed subject to touch all five dot‐surfaces during the matching phase. For each dot matrix, a 3D wireframe model was created and computer‐rendered in Adobe Photoshop to create a set of matching visual stimuli. Two distant light sources following the direction of the viewpoint provided lighting for each stimulus in such a way that all faces of the model were illuminated. Visual stimuli were displayed centrally on a gray background and subtended by a 5.0° visual angle. Corresponding to the haptic stimuli, the interdot spacing ranged from 0.1° to 0.9° and increased in steps of 0.2°.

### 2.3 Procedures

Within each functional run, there were four conditions of interest corresponding to a 2 × 2 design with modal (unimodal or crossmodal) and matching modality (visual or haptic) as independent factors. Stimulus timing and presentation were controlled using Presentation software (Neurobehavioral Systems, Inc.). Subjects laid supine in the MRI tunnel with earplugs and were instructed to relax. Subjects were asked to fixate on a white cross (viewing angle, 1.0° × 1.0°) that was projected from a projector through a mirror mounted to the head coil onto a screen. Each subject's right arm was extended to the top of the cover plate and comfortably supported by cushions. Each subject's left hand held the response box comprising four buttons. Subjects were asked to press the response buttons related to the task. The presentation of haptic stimuli was controlled by the experimenter who stood next to the bore scanner during the fMRI scans. Auditory cues delivered via headphones instructed the experimenter to adjust the haptic presentation plate to the correct stimulus during the interstimulus intervals. Prior to the initiation of the fMRI experiment, all subjects were trained to estimate the dot‐surfaces outside of the MR scanner until they felt comfortable performing the task.

#### 2.3.1 Unimodal condition: haptic‐to‐Haptic condition (hH)

As shown in Figure 1c, trial onset was cued by instructions in Chinese characters (haptic) for the first 2 s. Next, a haptic dot‐surface was presented for the 4 s encoding phase. Subjects were asked to explore the haptic dot‐surface by moving their right index finger and remember it while simultaneously fixating on the green visual cross presented on the screen. After the 2 s delay phase, matching phase onset was cued by instructions for 2 s. During the 10 s matching phase, subjects were asked to fixate on a blue visual cross and use to their right index finger to perceive all five dot‐surfaces. When the visual cross changed its color to red, subjects were asked to stop the movement of the right index finger and press the response button using their left hand to report which of the five stimuli was most similar in texture to the dot‐surface encoded during the encoding phase; this phase lasted 2 s. The total duration of one hH condition trial was 22 s.

#### 2.3.2 Unimodal condition: visual‐to‐Visual condition (vV)

As shown in Figure 1d, for the vV condition, we used the same procedure as that used in the hH condition. Since the object processing of the visual system is faster than that of a haptic system, we reduced the duration for visual stimuli presentation to keep both visual and haptic surface encoding at a similar level. The trial onset of the vV condition was cued by instructions in Chinese characters (visual) for the first 2 s. Next, a visual stimulus was presented for the 2 s encoding phase. Subjects were asked to look at the visual stimulus and remember it. After the 2 s delay phase, matching phase onset was cued by instructions for 2 s. During the 4 s matching phase, five visual stimuli were simultaneously presented with corresponding numbers, and subjects were asked to look at all five stimuli. When the visual stimuli disappeared and a red cross was presented, subjects were asked to press the response button to report which of the five stimuli was most similar in texture to the stimulus encoded during the encoding phase; this phase lasted 2 s. The total duration of one vV condition trial was 14 s.

#### 2.3.3 Crossmodal condition: haptic‐to‐Visual condition (hV) and visual‐to‐Haptic condition (vH)

The procedures of these two crossmodal conditions were the same as those of the unimodal conditions. However, in the hV condition, one haptic dot‐surface was presented during the encoding phase, and five visual stimuli were presented during the matching phase. Subjects were then asked to find a stimulus in the visual matching phase similar to that of the haptic stimulus. In contrast, in the vH condition, one visual stimulus was presented on the screen during the encoding phase and five haptic dot‐surfaces were presented during the matching phase. The subjects were then asked to find a stimulus in the haptic matching phase similar to that of the visual stimulus. The total durations of one hV trial and one vH trial were 16 and 20 s, respectively.

Each condition was repeated 25 times over the time course of the experiment. A total of 100 trials were randomly split into five functional runs.

### 2.4 Data acquisition

The crossmodal visuo‐haptic dot‐surface‐matching fMRI experiment was performed using a Siemens MAGNETOM Verio 3T scanner (Siemens). Standard sequence parameters were used to obtain functional images as follows: T2*‐weighted echo‐planar imaging; repetition time, 2,000 ms; echo time, 25 ms; flip angle, 77°; matrix, 64 × 64; 33 axial slices; field of view, 192 × 192 mm; thickness, 3.0 mm with a 0.6 mm interslice gap that covered the whole brain; and in‐plane resolution, 3.0 × 3.0 mm. After functional image acquisition, T1‐weighted high‐resolution anatomical images were obtained (voxel size, 0.97 × 0.97 × 1.0 mm^3^).

### 2.5 Univariate fMRI analyses

We used the Statistical Parametric Mapping (SPM12) package (Friston et al., 2007) implemented in MATLAB 7.5 (MathWorks) to process and analyze the fMRI data. The first four scan volumes of each fMRI run were discarded due to unsteady magnetization. Functional images from each run were realigned to the first volume of the first run and then realigned to the mean image after the first realignment. Slice‐timing correction was performed to adjust for differences in slice‐acquisition times. The T1‐weighted anatomical image was coregistered to the mean of all realigned images. Each coregistered T1‐weighted anatomical image was normalized to Montreal Neurological Institute (MNI) space using the DARTEL procedure (Ashburner, 2007). The parameters from the DARTEL procedure were then applied to each functional image and T1‐weighted anatomical image. The normalized functional images were filtered using a Gaussian kernel of 8‐mm FWHM in the *x*, *y*, and *z*‐axes. The parameters from this normalization process were then applied to the functional images, which were resampled to a final resolution of 2 × 2 × 2 mm^3^.

#### 2.5.1 Initial individual analysis

A general linear model was fitted to the fMRI data for each subject. The blood‐oxygen‐level‐dependent (BOLD) signal for all conditions was modeled with boxcar functions convolved with the canonical hemodynamic response function. For the experiment, the design matrix of each subject included the five functional runs, each of which included 20 regressors for the visual instructions (two) and encoding phase (four), matching phase (four), and response phase (four) for each trial. Furthermore, the motion‐related artifacts were minimized *via* the incorporation of six parameters (three displacements and three rotations) from the rigid‐body realignment stage into each model. The time series for each voxel was high‐pass filtered at 1/128 Hz. Assuming a first‐order autoregressive model, serial autocorrelation was estimated from the pooled active voxels with the restricted maximum‐likelihood procedure and used to whiten the data (Friston et al., 2002). The estimates were evaluated using the linear contrasts of each encoding phase (visual and haptic) and matching phase (hH, vV, hV, and vH) relative to rest in each subject. The obtained contrast images were then used for random‐effects group analysis.

#### 2.5.2 Random‐effects group analysis

To confirm brain activation during the haptic and visual encoding phases, we performed a one‐sample *t* test for each contrast. The height threshold for SPM{*t*} was set at *t* (35) = 3.44 (equivalent to *p* < .001, uncorrected). The statistical threshold for the spatial extent test on the clusters was set at *p* < .05, and the familywise error rate (FWE) was corrected for multiple comparisons over the whole brain.

Next, we employed a full factorial design to construct a single design matrix involving the matching phase of the hH, vV, vH, and hV conditions. All conditions were modeled as within‐subject (dependent) designs, and we evaluated the linear contrasts of these conditions. The height threshold for SPM{*t*} was set at *t* (68) = 3.21 (equivalent to *p* < .001, uncorrected). The statistical threshold for the spatial extent test on the clusters was set at *p* < .05, and the FWE was corrected for multiple comparisons over the whole brain. Coordinates in MNI space were labeled according to probabilistic maps (Eickhoff et al., 2005) in MNI space or the Talairach atlas after coordinate transformation into Talairach space (Lancaster et al., 2000, 2007). We initially identified the activation maps for regions involved in the haptic (hH > rest, vH > rest) and visual (vV > rest, hV > rest) matching phases. Then, we directly compared brain activity during crossmodal conditions with that during unimodal conditions (vH > hH and hV > vV) to identify brain regions involved in crossmodal processing. We subsequently conducted region of interest (ROI) analysis and used SPM12 to extract the BOLD signal from 8‐mm‐diameter spheres centered on the peak coordinates of all crossmodal specific regions.

### 2.6 Seed‐based resting‐state fMRI functional connectivity analysis

Resting‐state brain activation is widely recognized; brain regions that show good temporal correlation at rest are thought to functionally communicate over time, that is, functional connectivity (Kundu et al., 2012, 2013). Since previous studies have demonstrated that the network quantified at rest is significantly associated with the functional network of the same areas during a task, we confirmed seed‐based functional connectivity of the ROIs mentioned above to support our findings. An independent resting‐state dataset including 137 subjects (age range, 18–43 years) was adapted. Data were first processed by multi‐echo independent component analysis (ME‐ICA) using the tool *meica.py* as distributed in the Analysis of Functional NeuroImages (AFNI) neuroimaging suite (Cox, 1996) to select functionally related BOLD‐independent components and count the BOLD degrees of freedom (Kundu et al., 2012, 2013). Next, independent coefficient regression (ICR) was used to estimate seed‐based functional connectivity for each ROI. For a given subject‐level dataset, after computing the Pearson correlation (*r*) between independent coefficient vectors of all target voxels and the seed voxel, an ICR r‐map was produced. The *r* values were then converted to standard (*Z*) scores using the Fisher transform, which for ICR includes the standard error term to normalize the transformation for the number of functional BOLD components detected by ICA. Thus, a group‐level, seed‐based connectivity map was produced by simply conducting a one‐sample *t* test, voxelwise, on all subject‐level ICR Z‐maps using AFNI. Here, we conducted one‐sample *t* tests for three seed regions: left SPL (peak: −26, −47, 54), IPS (peak: −35, −58, 40), and IPL (peak: −38, −66, 34). For all subject‐level ICR Z‐maps, we set the threshold of the *t*‐statistic above 4.494 (*p* < .001/subject number n, *n* = 137).

## 3 RESULTS

### 3.1 Behavioral results

**FIGURE 2:**
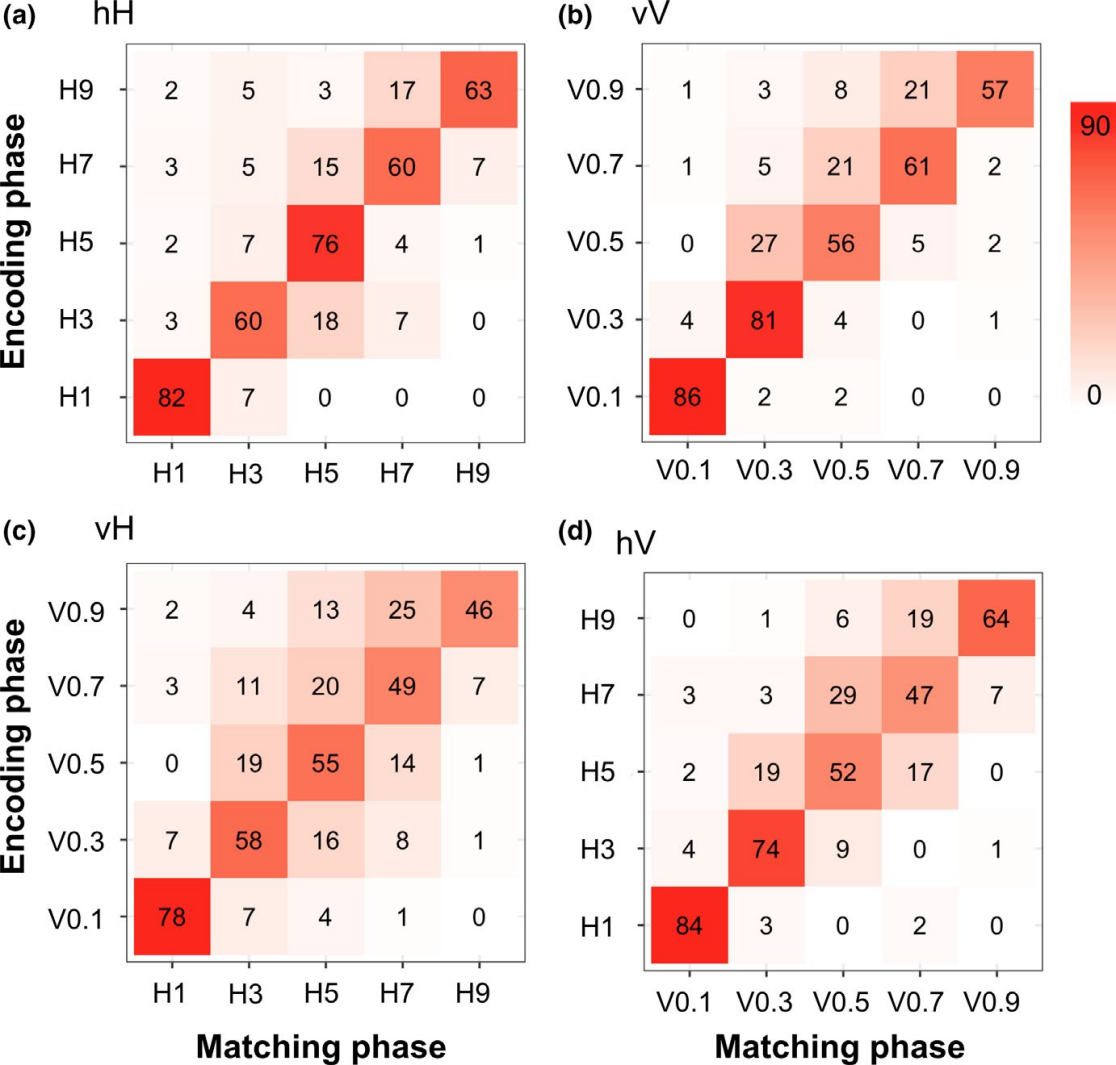
Confusion matrices of the responses obtained from the fMRI experiment. Each confusion matrix was derived from the pooled results of 18 subjects who participated in the fMRI experiment. Matrix entries represent the frequencies of all perceptual responses to each stimulus

Behavioral data were collected using Presentation software (Neurobehavioral Systems, Inc.). We used four 5 × 5 confusion matrices (Figure 2) to visualize the behavioral performance for all conditions. The confusion matrices are derived from the pooled results of the 18 subjects who participated in the fMRI experiment. Matrix entries represent the frequencies of all perceptual responses to each dot‐surface. Elements with red backgrounds on the main diagonal indicate that the subjects indicated the surface as presented. Briefly, we estimated the probability that the subjects would answer with the surface or the next most similar one. We then calculated the mean accuracies of all conditions (vV: 75.8 ± 3.5%; hH: 75.1 ± 4%; vH: 62.9 ± 3.9%; hV: 71.3 ± 3.1%), and all exceeded chance level (20%). A one‐way repeated measures analysis of variance was performed using R Studio (version 3.2.4), which identified a significant main effect of the condition [*F* (3, 51) = 4.367; *p* = .008]. Post hoc pairwise comparisons (with Bonferroni correction) indicated no significant differences among the hH, vV and hV conditions (*p*s > .05). By contrast, the same test indicated that the accuracy of the vH condition was significantly decreased compared with that of the vV (*p* = .008) and hH (*p* = .014) conditions.

### 3.2 Univariate fMRI results

#### 3.2.1 Regions involved in haptic and visual dot‐surface encoding processing

**FIGURE 3:**
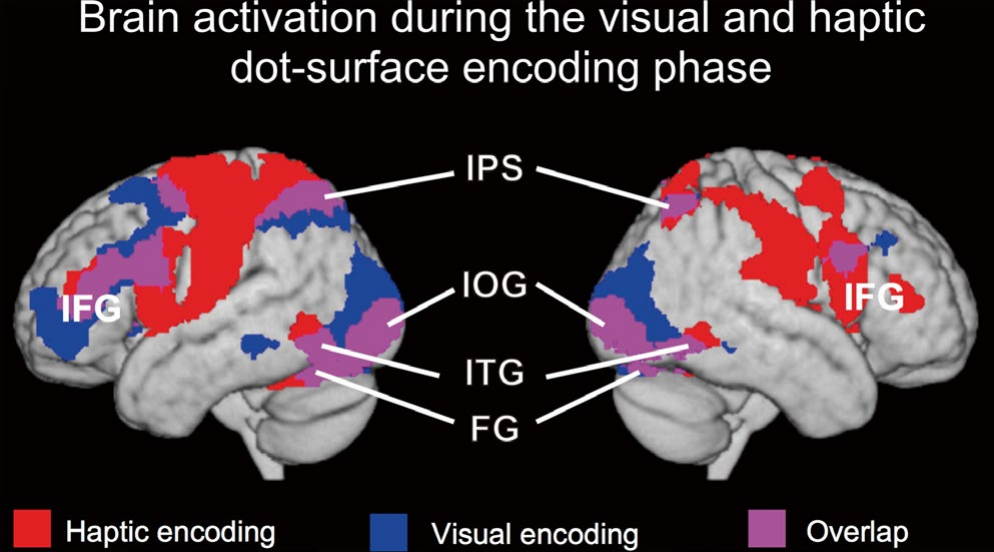
Brain activation during the visual and haptic dot‐surface encoding phase. Areas of significant activation are rendered on a normalized T1‐weighted high‐resolution brain MRI averaged across 18 subjects and the axial section of the same image. The extent threshold for activation was *p* < .05, corrected for each search volume with a height threshold of *T* (68) = 3.21 (equivalent to *p* < .001, uncorrected). FG, fusiform gyrus; IFG, inferior frontal gyrus; IOG, inferior occipital gyrus; IPS, intraparietal sulcus; ITG, inferior temporal gyrus

Figure 3 illustrates the brain activation regions of the haptic and visual encoding phases. The encoding processing of each modality activated a widespread set of brain regions. The common regions of both modalities (overlapping purple regions) included the bilateral inferior occipital gyrus (IOG), fusiform gyrus (FG), inferior frontal gyrus (IFG), inferior temporal gyrus (ITG), and bilateral IPS. In addition to these regions, visual encoding processing also activated more regions (blue regions), including the bilateral lingual gyrus (LG), middle occipital gyrus (MOG), and superior occipital gyrus (SOG). In contrast, haptic encoding processing activated more regions (red regions), including the bilateral postcentral gyrus (poCG), precentral gyrus (preCG), middle frontal gyrus (MFG), parietal operculum (PO), insula, SPL, and bilateral medial frontal gyrus (mFG).

#### 3.2.2 Regions involved in haptic and visual dot‐surface match processing

**FIGURE 4:**
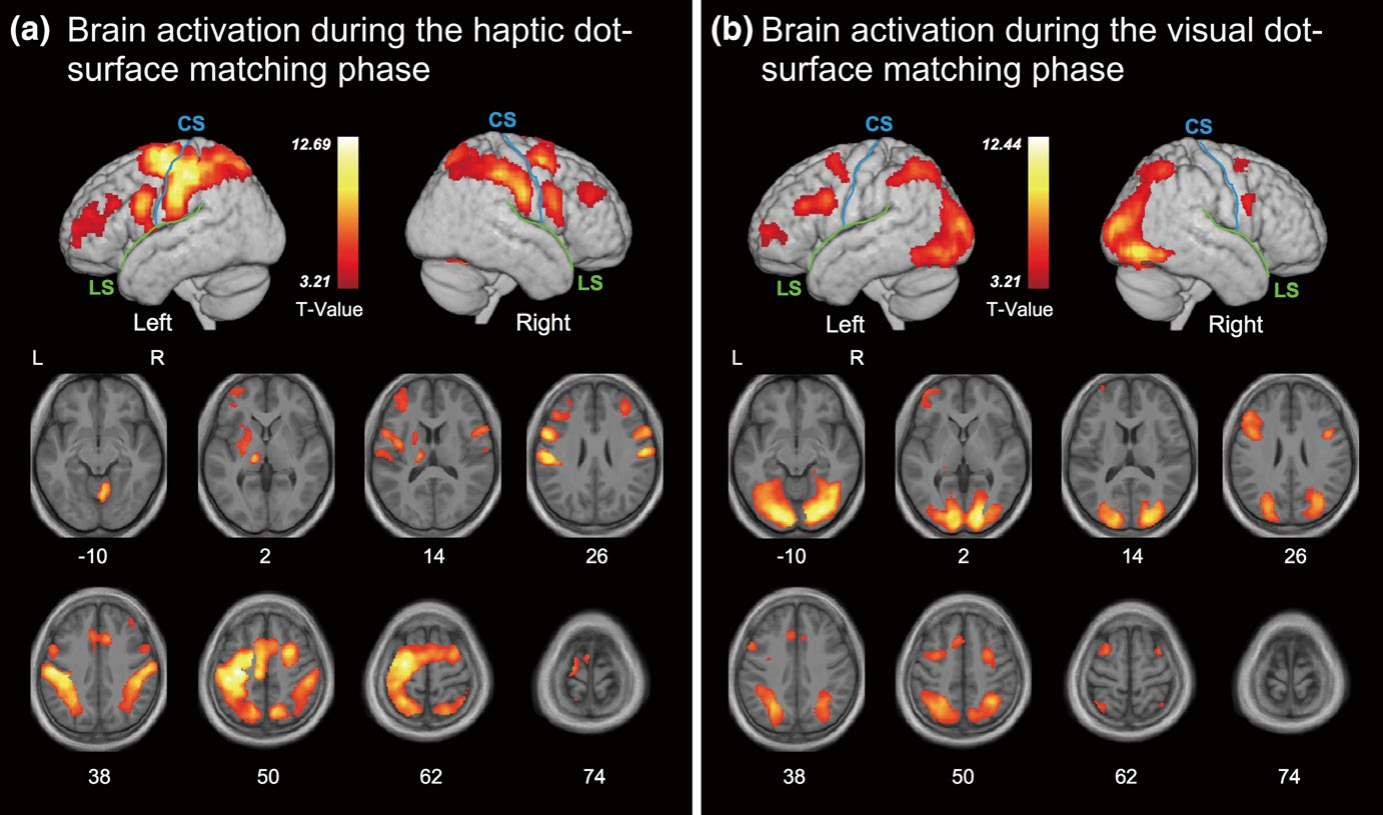
Brain activation patterns for haptic and visual dot‐surface match processing. (a) Common brain activation for the haptic dot‐surface‐matching phase. The results of the contrast (hH > rest) ∩ (vH > rest) are shown. (b) Common brain activation for the visual dot‐surface‐matching phase. The results of the contrast (vV > rest) ∩ (hV > rest) are shown. Areas of significant activation are rendered on a normalized T1‐weighted high‐resolution brain MRI averaged across 18 subjects and the axial section of the same image. The solid blue and green lines shown in the rendered images indicate the central sulcus (CS) and lateral sulcus (LS), respectively. The extent threshold for activation was *p* < .05, corrected for each search volume with a height threshold of *T* (68) = 3.21 (equivalent to *p* < .001, uncorrected)

In Figure 4a, we presented the common brain activation maps of unimodal and crossmodal haptic dot‐surface match processing using the (hH > rest) ∩ (vH > rest) contrast (Nichols et al., 2005). Furthermore, we also show the common brain activation maps of unimodal and crossmodal visual dot‐surface match processing using the (vV > rest) ∩ (hV > rest) contrast in Figure 4b. Specifically, haptic dot‐surface match processing revealed regions of significant activation in the bilateral IPL, SPL, IFG, MFG, angular gyrus, cingulate gyrus, insula, and precuneus, as well as the left PreCG, PoCG, mFG, and superior frontal gyrus (SFG). The contrast revealed additional regions of activation in the left claustrum, left lentiform nucleus, and right cerebellum. Evaluation of visual dot‐surface match processing revealed regions of significant activation in the bilateral LG, MOG, FG, SPL, and precuneus, as well as the left IFG, MFG, and cuneus gyrus.

#### 3.2.3 Specific regions for crossmodal (vH > hH and hV > vV) dot‐surface match processing

**FIGURE 5:**
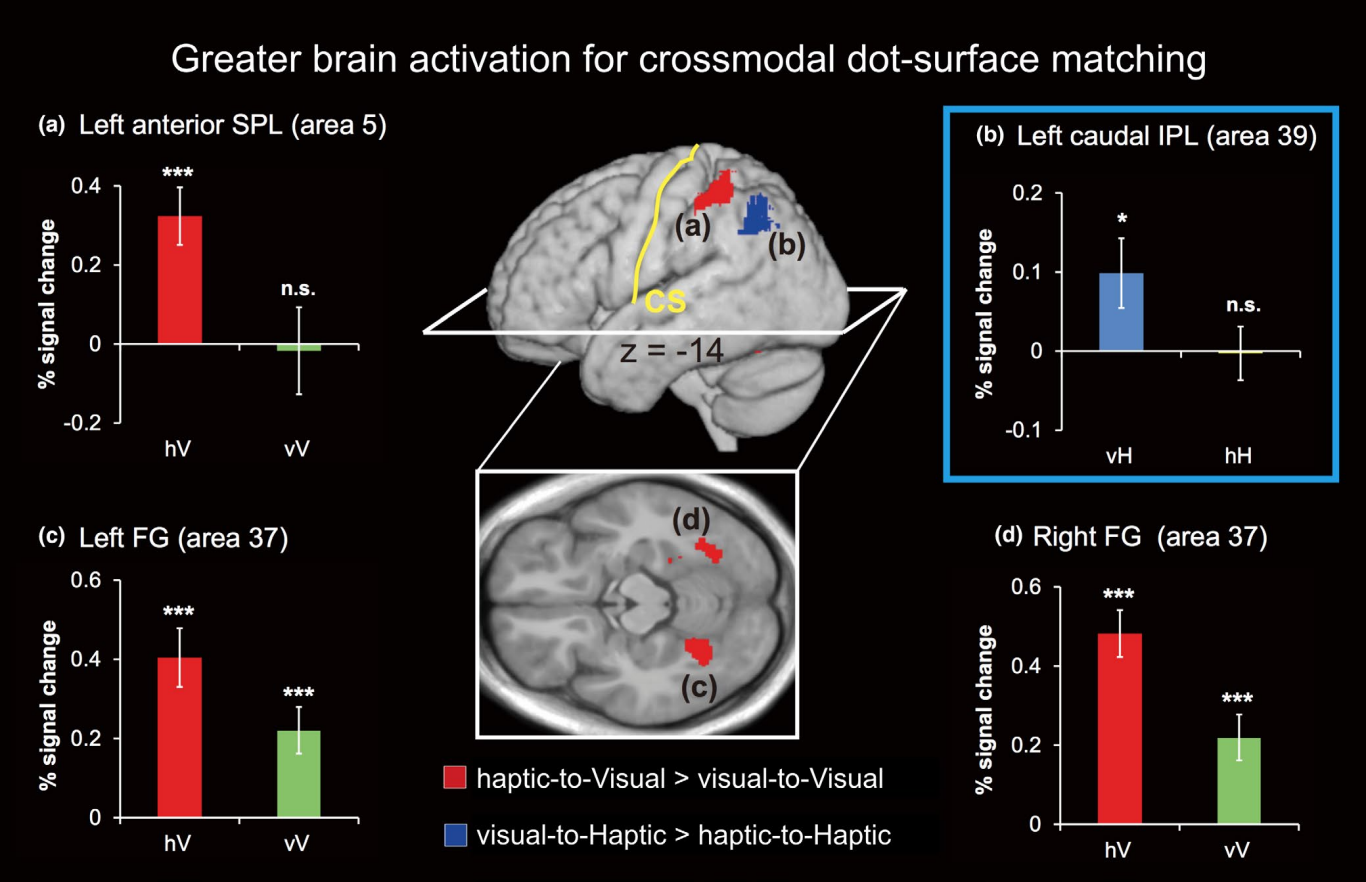
Greater brain activation for crossmodal conditions. Regions of significant activation are rendered on a normalized T1‐weighted high‐resolution brain MRI averaged across 18 subjects and the axial section of the same image. The extent threshold for activation was *p* < .05, corrected for each search volume with a height threshold of *T* (68) = 3.21 (equivalent to *p* < .001, uncorrected). The colored bar graphs of (a–d) indicate the percent BOLD signal change relative to rest by using a volume of interest with an 8‐mm‐diameter sphere. The centers of the spheres were the peak coordinates of activation. Data are presented as the mean ± *SEM* of 18 subjects. Asterisks represent regions that showed significant positive activation relative to rest (n.s., no significant difference; *p < .05; ***p < .001, one‐sample *t* test). FG, fusiform gyrus; IPL, inferior parietal lobule; poCG, postcentral gyrus; SPL, superior parietal lobule

To determine the crossmodal dot‐surface‐matching specific brain regions, we then compared data from the hV condition with data from the vV condition and data from the vH condition with data from the hH condition. As shown in Figure 5, the hV > vV contrast revealed significant activation in the bilateral FG (i.e., area 37) and left anterior SPL (i.e., area 5), whereas the vH > hH contrast revealed a significant activation only in the left caudal IPL (i.e., area 39). In Figure 5a–d, the colored bar graphs indicate the percent of BOLD signal change relative to rest in each region. We then conducted a one‐sample *t* test to address whether these regions showed significant positive activation compared with rest. The results suggest that the specific region of the left anterior SPL specific to the hV > vV condition showed a significant positive activation only in the hV condition, but the bilateral FG showed a significant positive activation in both the hV and vV conditions. Furthermore, vH > hH‐specific regions of the left caudal IPL were activated only for the vH condition.

### 3.3 Differences in resting‐state fMRI functional connectivity of three left LPPC subregions

**FIGURE 6:**
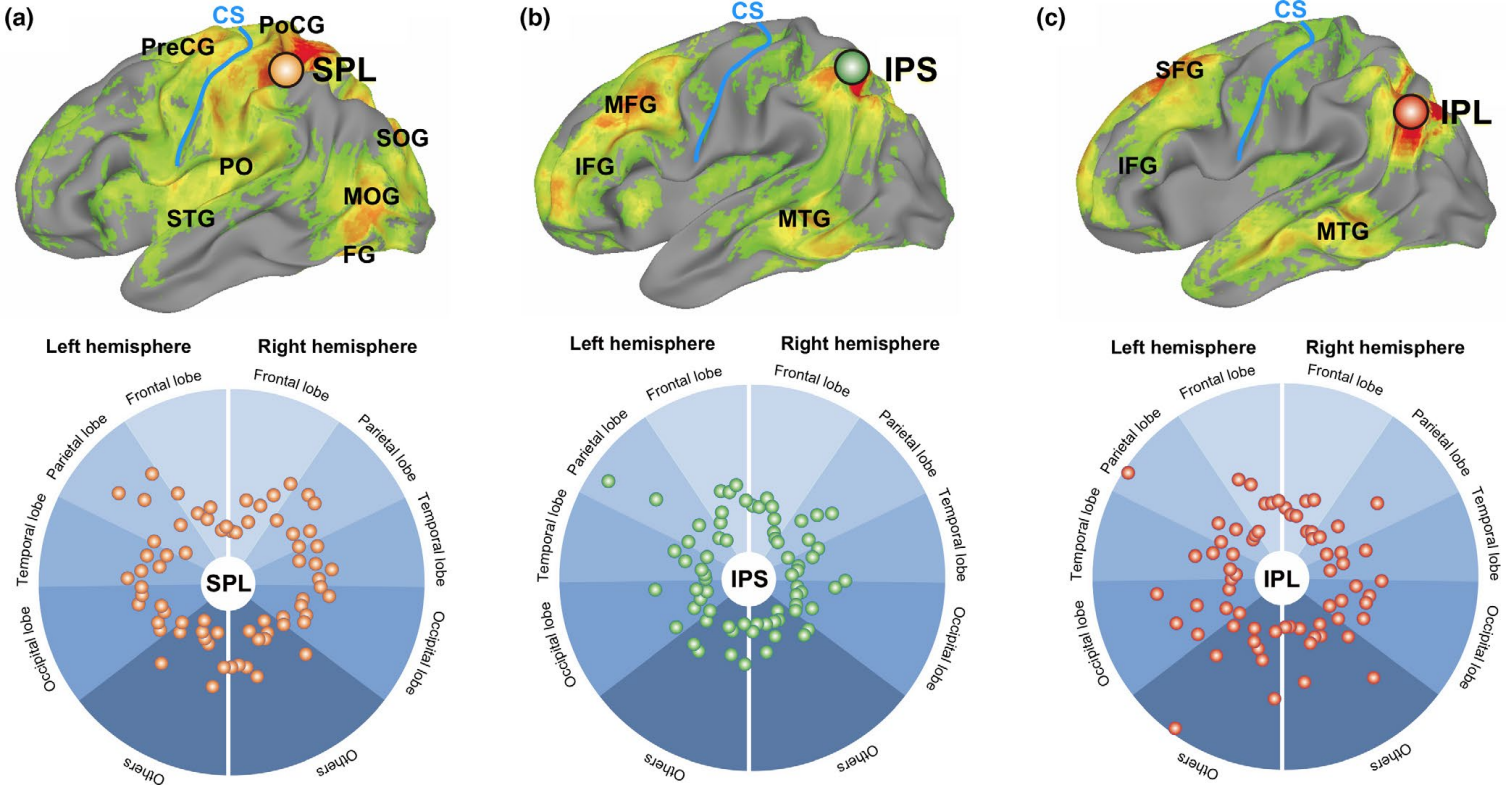
Resting‐state fMRI functional connectivity of three left LPPC subregions. We selected different seed regions (a: IPL, b: IPS, and c: SPL) to find the different seed‐based resting‐state networks. The dots in the radar charts of the bottom row show the mean *Z*‐scores (Pearson correlations were converted to *Z*‐scores using the Fisher transform) and range from 0 (center of the circle) to 0.4 (circumference). The group‐level seed‐based connectivity map was produced by conducting a one‐sample *t* test, voxelwise, on all subject‐level independent coefficient regression *Z*‐maps and setting the threshold of the *t*‐statistic above 4.494 (*p* < .001/subject number *n*, *n* = 137)

The functional networks defined from resting‐state fMRI are considered to be recruited and combined to perform tasks. To investigate how three left LPPC subregions, namely the SPL, IPS, and IPL, connect to other brain regions in a resting state, we estimated seed‐based resting‐state fMRI functional connectivity using an independent dataset. The dots in the radar charts in Figure 6 show the mean *Z*‐scores and range from 0 (center of the circle) to 0.4 (circumference). As shown in Figure 6, the left SPL was functionally correlated with a group of brain regions, including the bilateral PreCG, PoCG, PO, dorsal SPL, superior temporal gyrus (STG), SOG, MOG, and FG. The left IPS correlated network mostly included the bilateral IPS, IFG, MFG, and middle temporal gyrus (MTG). The left IPL correlated network showed partial overlap with the IPS correlated network, including the bilateral MTG and anterior IFG; however, correlation of the frontal cortex was mostly with the bilateral SFG.

## 4 DISCUSSION

In the present study, we investigated the brain network of crossmodal visuo‐haptic dot‐surface matching using an fMRI experiment. We found that the left LPPC showed functional heterogeneity during visuo‐haptic crossmodal dot‐surface retrieval. Specifically, our results revealed that haptic‐to‐visual crossmodal retrieval enhanced the activity in the left SPL relative to visual unimodal retrieval (Figure 5a), whereas the inverse visual‐to‐haptic condition enhanced the activity in the left IPL (Figure 5b). Unlike left SPL and IPL activation, left IPS activation did not show a prominent difference between crossmodal and unimodal retrieval. These differences are understandable in the context of different dot‐surface encoding properties existing between the visual and haptic systems (Klatzky & Lederman, 2010; Klatzky et al., 1987), thereby resulting in different crossmodal memory retrieval processing, which modulates the activation of left LPPC subregions. The subsequent seed‐based resting connectivity analysis provided additional evidence to support functional heterogeneity in the left LPPC during visual and haptic crossmodal dot‐surface matching by observing three left LPPC subregions engaging distinct networks (Figure 6).

Here, we confirmed that visual and haptic dot‐surface encoding processing shared a larger proportion of brain areas, including the bilateral IFG, IPS, IOG, ITG, and FG (Figure 3). On the one hand, bilateral IFG and IPS are involved in the frontal–parietal network, which contributes a wide variety of modality‐independent tasks (Kassuba et al., 2013; Kitada et al., 2006; Yang et al., 2014; Yu et al., 2018) and serves as a flexible hub of cognitive control (Zanto & Gazzaley, 2013). On the other hand, bilateral IOG, ITG, and FG are included in the ventral visual pathway, which is known to form object representations in the brain (Kravitz et al., 2013). Thus, these common areas are thought to functionally connect with modality‐specific areas such as the primary somatosensory cortex or the primary visual cortex to form dot‐surface representations in the human brain. For both visual and haptic dot‐surface match processing, we also found a similar activation pattern as the encoding phase for each modality (Figure 4a,b). Nevertheless, several additional functions, such as memory retrieval and decision making, are incorporated into these brain networks since one has recalled a specific detail of a stimulus from a past encoded event and compared it with a current stimulus. Furthermore, crossmodal match processing was expected to engage some areas included in the network, which should contribute to information transfer and/or convert the formation of the stimulus to match the different modalities.

We highlighted two crossmodal retrieval‐specific regions of the left LPPC (Figure 5a,b) by contrasting the brain activations of the matching phase of crossmodal to unimodal conditions. Specifically, the left SPL showed specificity for the hV condition compared to the unimodal vV condition, whereas the left IPL was more sensitive for the vH condition than for the unimodal hH condition. In other words, this finding suggests that visuo‐haptic crossmodal retrieval will engage different left LPPC subregions dependent on the dot‐surface information retrieval order (i.e., haptic‐to‐visual or visual‐to‐haptic). This finding may reflect functional heterogeneity in the left LPPC during crossmodal retrieval processing. According to previous findings, the left LPPC is a highly heterogeneous region that is anatomically and functionally connected to the prefrontal cortex (Borra & Luppino, 2017; Nelson et al., 2010), and it plays an important role in sensory and cognitive processing, including spatial perception and memory retrieval (Cabeza et al., 2008). In particular, the left IPL mostly contributes to the specific object feature recollection from the encoding phase (Sestieri et al., 2017), whereas the SPL contributes to the manipulation and rearrangement of information in working memory (Koenigs et al., 2009). Thus, in the present study, left IPL activation may reflect the recollection of the encoded visual dot‐surface and matching it to the haptic surface. In contrast, left SPL activation is thought to contribute to rearranging the encoded haptic dot‐surface to match the visual surface.

One possible interpretation of the left LPPC functional heterogeneity for crossmodal retrieval is related to the difference in object encoding strategies between the visual and haptic systems. Given the salience of haptic texture encoding (Klatzky & Lederman, 2010; Sathian, 2016), touch is thought to extract more specific surface properties than visual encoding. In contrast, the visual system should give greater weight to the spatial pattern during dot‐surface encoding. Thus, this difference may reflect the direct influence of the perceptual representation of dot‐surfaces in the brain for different modalities. In line with this view, it is reasonable to assume that the human brain has to rearrange or convert the encoded haptic surface substance to match the incoming visual–spatial pattern. For the inverse case, one is more likely to extract the spatial pattern of the haptic dot‐surface during the matching phase and recollect the specific details of the encoded visual dot‐surface to match it. This assumption can also explain why we observed more significant bilateral FG activations for the hV condition, which contribute to the conversion of the dot‐surface from “haptic space” to “visual space” (Masson et al., 2016).

Finally, we observed distinct resting‐state functional connectivity networks for these three left LPPC subregions (Figure 6), which supports our view of LPPC functional heterogeneity during crossmodal memory retrieval. Spontaneous brain activity during rest has been studied in humans for more than two decades, and resting‐state functional connectivity networks have been shown to specifically correlate with task‐driven networks (Fox & Raichle, 2007; Hermundstad et al., 2013; Shen, 2015). One possibility is that resting‐state functional connectivity networks represent the type of regions likely to be used in future tasks. Thus, we used seed‐based, resting‐state functional connectivity analysis to confirm whether these three left LPPC subregions are functionally connected with different regions. As shown in Figure 6, we found that the left SPL was strongly connected to bilateral somatosensory areas and higher visual areas (e.g., V2, V3), which have been implicated in high‐level haptic and visual information processing. This correlation pattern is similar to the activation pattern of visual and haptic dot‐surface encoding observed in the present study. In contrast, the network containing the left IPL consisted of regions, including the bilateral MFG, frontal eye fields, ITG, and MTG, which are responsible for saccadic eye movements for visual field perception and awareness. In particular, the bilateral ITG/MTG in the ventral visual pathway shows a clear functional role in visual memory retrieval (Takeda, 2019). However, the left IPS was strongly connected to the bilateral prefrontal cortex and ITG/MTG. These regions have been implicated in planning, complex cognitive behaviors, attention, and decision making (Hunt et al., 2018; Nee & D'Esposito, 2016; Tremel & Wheeler, 2015), rather than haptic or visual object perception per se.

## 5 CONCLUSION

In summary, to explore LPPC functional heterogeneity between unimodal memory retrieval and crossmodal memory retrieval, we used a crossmodal visuo‐haptic dot‐surface‐matching fMRI task. Completing this task required subjects to remember the dot‐surface during the encoding phase, maintain this information during the delay phase, and find the same (i.e., unimodal conditions) or a similar (i.e., crossmodal condition) stimulus during the matching phase. When we focused on the matching phase, our design allowed us to test the neural substrates of the crossmodal dot‐surface comparison. From the standpoint of memory retrieval, our findings have provided the knowledge that left LPPC subregions do not simply contribute to the retrieval of past information; rather, each subregion has a more specific functional role in resolving different task requirements. This finding suggests that activity in the left LPPC cannot be easily explained by a singular processing pathway. Further studies are thus required to determine the specific roles of LPPC subregions in crossmodal memory retrieval processing.

## CONFLICT OF INTERESTS

All authors declare that they have no other competing interests.

## AUTHOR CONTRIBUTIONS

J.Y., Y.Y., H.S., H.K., and K.N. designed and performed the fMRI experiments. Y.E. and J.W. contributed to the conception and design. J.Y., Y.Y., and T.K. analyzed the fMRI data. J.Y. and Y.Y. wrote the paper. All authors discussed and commented on the manuscript.

## Data Availability

The data presented here are available from the corresponding author upon reasonable request.
